# State-of-the-art of lumbar puncture and its place in the journey of patients with Alzheimer’s disease

**DOI:** 10.1002/alz.12372

**Published:** 2021-05-27

**Authors:** Harald Hampel, Leslie M. Shaw, Paul Aisen, Christopher Chen, Alberto Lleó, Takeshi Iwatsubo, Atsushi Iwata, Masahito Yamada, Takeshi Ikeuchi, Jianping Jia, Huali Wang, Charlotte E. Teunissen, Elaine Peskind, Kaj Blennow, Jeffrey Cummings, Andrea Vergallo

**Affiliations:** 1 Eisai Inc., Neurology Business Group, Woodcliff Lake, New Jersey, USA; 2 Perelman School of Medicine, Department of Pathology and Laboratory Medicine, University of Pennsylvania, Philadelphia, Pennsylvania, USA; 3 USC Alzheimer’s Therapeutic Research Institute, San Diego, California, USA; 4 Memory Aging and Cognition Centre, Departments of Pharmacology and Psychological Medicine, Yong Loo Lin School of Medicine, National University of Singapore, Singapore; 5 Sant Pau Memory Unit, Hospital de la Santa Creu i Sant Pau–Biomedical Research Institute Sant Pau–Universitat Autònoma de Barcelona, Barcelona, Spain; 6 Centro de Investigación Biomédica en Red de Enfermedades Neurodegenerativas (CIBERNED), Instituto de Salud Carlos III, Madrid, Spain; 7 Department of Neuropathology, Graduate School of Medicine, The University of Tokyo, Tokyo, Japan; 8 Tokyo Metropolitan Geriatric Hospital, 35-2 Sakaecho, Itabashi-ku, Tokyo, Japan; 9 Department of Neurology and Neurobiology of Aging, Kanazawa University Graduate School of Medical Science, Kanazawa, Japan; 10 Department of Molecular Genetics, Brain Research Institute, Niigata University, Asahimachi, Niigata, Japan; 11 Innovation Center for Neurological Disorders, Department of Neurology, Xuan Wu Hospital, Capital Medical University, Beijing, China; 12 Dementia Care and Research Center, Peking University Institute of Mental Health (Sixth Hospital), Beijing Dementia Key Lab, National Clinical Research Center for Mental Disorders, Beijing, China; 13 Neurochemistry Laboratory, Department of Clinical Chemistry, Amsterdam Neuroscience, Vrije Universiteit Amsterdam, Amsterdam UMC, Amsterdam, the Netherlands; 14 VA Northwest Mental Illness Research, Education and Clinical Center, VA Puget Sound Health Care System, Department of Psychiatry and Behavioral Sciences, University of Washington School of Medicine, Seattle, Washington, USA; 15 Department of Psychiatry and Neurochemistry, The Sahlgrenska Academy at the University of Gothenburg, Mölndal, Sweden; 16 Clinical Neurochemistry Laboratory, Sahlgrenska University Hospital, Gothenburg, Sweden; 17 Chambers-Grundy Center for Transformative Neuroscience, Department of Brain Health, School of Integrated Health Sciences, University of Nevada Las Vegas (UNLV), Las Vegas, Nevada, USA

**Keywords:** Alzheimer’s disease, biomarker, cerebrospinal fluid, diagnosis, evidence-based guidelines, lumbar puncture, system readiness

## Abstract

Recent advances in developing disease-modifying therapies (DMT) for Alzheimer’s disease (AD), and the recognition that AD pathophysiology emerges decades before clinical symptoms, necessitate a paradigm shift of health-care systems toward biomarker-guided early detection, diagnosis, and therapeutic decision-making. Appropriate incorporation of cerebrospinal fluid biomarker analysis in clinical practice is an essential step toward system readiness for accommodating the demand of AD diagnosis and proper use of DMTs—once they become available. However, the use of lumbar puncture (LP) in individuals with suspected neurodegenerative diseases such as AD is inconsistent, and the perception of its utility and safety differs considerably among medical specialties as well as among regions and countries. This review describes the state-of-the-art evidence concerning the safety profile of LP in older adults, discusses the risk factors for LP-associated adverse events, and provides recommendations and an outlook for optimized use and global implementation of LP in individuals with suspected AD.

## 1 | INTRODUCTION

**FIGURE 1:**
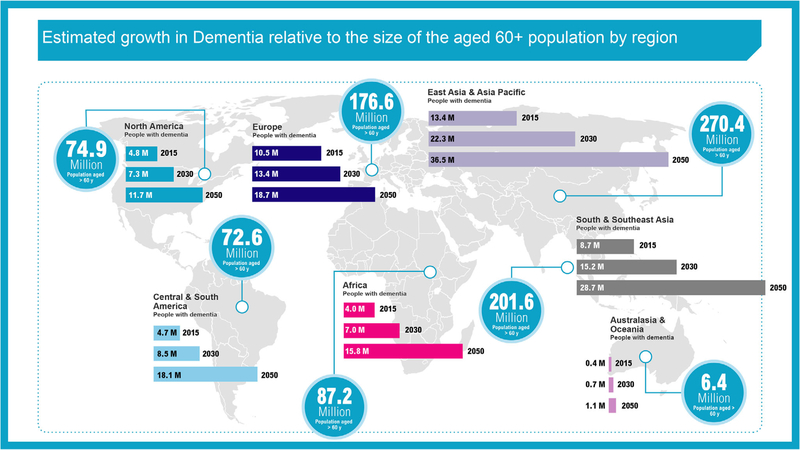
Estimated growth in dementia relative to the size of the aged 60+ population by region. Global incidence and projected growth in numbers of people living with dementia^2^

Alzheimer’s disease (AD) is a multifactorial neurodegenerative disease that results from complex interactions among genetic, biological, and environmental factors. Most AD cases are sporadic, and disease risk increases with age. Epidemiological projections indicate that AD is a global and fast-growing public health epidemic. Worldwide, the proportion of people aged 65 years or older is growing rapidly, and it is estimated that by 2060, this age group will account for ≈24% to 29% of the population in Western Europe and the United States, and 29% to 40% of the population in Asian countries (including China, Japan, Singapore, South Korea, and Taiwan).^1^ With the increase in longevity and aging populations, the population burden of age-related neurodegenerative diseases such as AD will rise significantly. Worldwide, an estimated 46.8 million people were living with all forms of dementia in 2015, and this number is expected to reach 131.5 million in 2050 (Figure 1).^2^

AD is the most common cause of dementia, accounting for ≈60% to 80% of cases.^3^ From a biological standpoint, roughly 30% of clinically normal individuals 65 years of age and older have biomarker evidence of amyloid accumulation, placing them on the AD biological continuum.^4–6^ With the progress in understanding AD pathophysiology and the current definition of AD as a clinical biological framework,^7^ a number of candidate drugs targeting distinct molecular pathways such as the amyloid beta (A*β*) pathway have shown putative disease-modifying effects and have reached late stages of clinical development for the treatment of patients in early stages of the AD clinical continuum, including the mild cognitive impairment (MCI) stage (i.e., prodromal AD), as well as for secondary prevention of cognitive impairment and dementia in patients with preclinical AD.

### 1.1 | The challenges facing systems preparedness for AD-modifying therapies

If a therapeutic approach with a demonstrated effectiveness in slowing the biological and clinical progression of AD were to become available, at least 20 million people in the European Union^8^ and 15 million in the United States^9^ would require timely and systematic biological screening and diagnostic work-up. Beyond diagnosis, biomarkers will play a key role for prognostic evaluation and screening, which are essential for both pharmacological clinical trials and future medical practice.^10^ In the clinical trial setting, biomarkers are used for patient selection, target engagement, dose adjustment, and long-term identification and monitoring of biological effects.^7,11^

Extensive evidence indicates that prevention and early intervention is the most effective way to combat dementia and preserve brain health at the highest functional level.^12,13^ It is estimated that the US health-care system could save $7.9 trillion if all AD were diagnosed early during the MCI stage rather than at the late, full-blown dementia stage.^3^ To prepare health-care systems for disease-modifying therapies (DMTs) for AD, building capacities for systematic assessment of AD pathophysiological biomarkers, such as those in the evolving amyloid/tau/neurodegeneration (AT[N]) system,^7^ is imperative.

### 1.2 | The utility of cerebrospinal fluid biomarkers for future clinical practice

Two well-validated modalities, cerebrospinal fluid (CSF) biomarkers and positron emission tomography (PET), have been widely used to assess AD pathophysiology in vivo.^14–16^ Blood-based biomarkers represent a promising direction of AD biomarker research and hold enormous potential to transform clinical practice, because blood tests are globally accessible and cost-, resource-, and time-effective. However, at the current stage, blood-based biomarkers are still under development, and both analytical validation and standardization efforts as well as much more research is needed to establish their utility in clinical trials and clinical practice. Comparing the two more established modalities, while PET offers unique insights into the spatial and temporal evolution of AD pathophysiology, CSF analysis is more cost-effective, is less resource-intense, provides evidence on several pathophysiological processes, and is more accessible globally compared to PET brain imaging.^8,9^ The number of PET scanners and cyclotrons is limited, and existing scanners likely have limited time-slots to accommodate additional patients who need A*β* and/or tau PET assessments. More importantly, one single CSF analysis with fully automated assays can allow simultaneous investigation of all of the AT(N) biomarkers (Table 1), thus dramatically accelerating diagnostic, prognostic, and therapeutic decision-making.^17–21^

After decades of development and optimization, several well-validated CSF biomarkers show strong and consistent association with AD, and thus have been integrated into the research diagnostic criteria for AD.^7,16^ A comprehensive summary of the diagnostic and prognostic value of CSF biomarkers is beyond the scope of the current article, and can be found in recent reviews.^22–24^

### 1.3 | Factors that limit the widespread use of lumbar puncture and CSF biomarker assessment in AD diagnosis

Despite these advantages, CSF biomarker analysis in the context of AD—especially its application at suspected preclinical or prodromal stages—has in many countries been hampered partially by low rates of recommendation and performance of lumbar puncture (LP; also called spinal tap), the medical procedure for CSF collection.^25,26^ Regional differences in medical practice, insurance coverage, and clinical guidelines influence the number of LPs performed each year in individuals with suspected AD pathophysiology. Scandinavian and several other European countries have relatively high rates of performance of LP for AD diagnostics in clinical practice, whereas in North America, the use of LP is largely limited to academic clinical and research settings.^27^ The overall landscape analysis indicates a considerable hesitation in requesting LP for AD. This is highlighted by a recent analysis of nationwide healthcare insurance claims data in Germany, which reported that <1% of patients with MCI or overt dementia underwent LP and CSF analysis.^28^ Such hesitation may be attributable to the perceived invasiveness of the LP procedure and potential adverse events (AEs), especially when serial procedures are required, and with limited utility of early diagnosis and limited improvement from currently available AD therapies. The concerns about safety are particularly important in the case of prodromal stages of AD (and in the future also for preclinical stages of AD). Last but not least, physicians’ lack of confidence and experience in performing LP and CSF biomarker analysis, as well as the perception that diagnosis is not that important in the absence of an effective treatment, are all likely to play a role in limiting the use of this procedure.

To examine the incidence and prevalence of AEs occurring during and after LP performed in the context of an MCI or dementia syndrome, international research frameworks and workgroups have conducted prospective studies, as well as systematic registry- and literature-based reviews and meta-analyses.^25,26,28–34^ While these studies have not been double-blinded, or even blinded for the patient, this effort has generated evidence-based medical guidance that supports and informs the more widespread use of LP in AD clinical practice and clinical trial settings.^31–33^ In addition, informative videos to demonstrate the procedure to physicians and to inform patients, based on the international consensus guidelines, have been developed and are published with open access.^35,36^

Here we provide a state-of-the-art review of the most recent and relevant evidence about the safety and tolerability of contemporary LP when applied to neurodegenerative diseases, particularly in the groups most likely to be investigated and treated for AD. In addition, we review the evidence-based clinical guidance and recommendations for the optimized use of LP in individuals with suspected AD pathophysiology. We highlight new and emerging techniques that hold the potential to significantly improve the safety and feasibility of LP by tailoring the performance of the procedure to the patient’s anatomical and medical profiles. Finally, we review some of the main challenges to be overcome to facilitate implementing large-scale, CSF-based diagnostic and therapeutic decision-making paradigms, which would ultimately enable timely management of AD on a global scale.

## 2 | SAFETY OF LP IN SUBJECTIVE COGNITIVE DECLINE, MCI, AND AD

### 2.1 | An overview of the most relevant clinical evidence

The currently available medical evidence shows that LP is a safe procedure in older adults with or without cognitive impairment.^31^ The safety profile of LP has been comprehensively documented in studies leveraging clinical trial data, involving >7000 patients, as well as in routine clinical practice, involving >30,000 patients with a variety of neurological disorders.^33^ In the context of AD, multiple retrospective and prospective studies indicate that LP can be performed safely with low complication rates in patients with suspected AD (<1% of serious complaints requiring specialist treatment).^25,26,29,31–34^ Details of key studies discussed below are summarized in Table 2.

### 2.2 | Rates of adverse events

The largest prospective multicenter feasibility study in the population of interest involved 3868 patients who attended memory clinics, of whom 3558 underwent LP, and of whom 3456 were available for follow-up assessment. In the total study population, 20% of participants had subjective cognitive decline, 25% had MCI, 40% were diagnosed with dementia, and the remaining individuals were diagnosed with other neurological or psychiatric diseases.^29^ The mean (standard deviation [SD]) age was 66 (11) years, and the mean (SD) Mini-Mental State Examination score was 25 (5). This study population was representative of a real-world hospital or outpatient practice. One thousand sixty-five participants (31%) reported post-LP complaints, and far fewer individuals required medical intervention. Specifically, 11 patients (0.3%) received an epidural blood patch, and 23 patients (0.7%) required hospitalization for medical monitoring. All patients had complete recovery after treatment. Based on these findings, the authors concluded that LP can be performed safely in patients undergoing a diagnostic work-up for suspected AD, and that the knowledge of risk factors can be leveraged to reduce the prevalence of complaints. In the Japanese Alzheimer’s Disease Neuroimaging Initiative study, 198 of the total 537 individuals (36.9%) across the mild AD, late MCI, and cognitive normal groups had LP, without any serious AEs reported.^37^

### 2.3 | Adverse events profiles after LP

LPs may be associated with specific AEs, which are generally mild and manageable. Headache and back pain are the most commonly reported post-LP AEs. According to The International Classification of Headache Disorders third edition, post-dural puncture headache is described as headache occurring within 5 days of a LP, believed to be caused by CSF leakage through the dural puncture.^38^ Typically, it develops within 3 days of the procedure and manifests as an orthostatic or postural, usually frontal, headache, precipitated by moving from a supine to an upright position and resolving within 20 seconds of recumbence.^31,34^ The incidence of typical post-LP headache ranges from 0.9% to 9.0%.^26,29,32,34^ More than 85% of post-LP headaches resolve without treatment. While the pathophysiological dynamics of post-LP headache are not fully elucidated, in-human data indicate that the leakage of CSF leads to transient intracranial hypotension (increased by standing), which causes dilation of as well as pressure and traction on intracranial veins, eventually triggering meningeal nociceptive terminals.^39,40^

While mild cases of headache can be treated with caffeine or paracetamol/caffeine, the management of severe and persistent headache usually involves the use of an epidural blood patch,^31^ a procedure, although rarely used in older individuals, that has been shown to be effective (leading to complete recovery) and well-tolerated in all age ranges, including patients over 65 years old.^41^ The clinical spectrum of headache after an LP procedure may vary, and besides the typical post-LP headache, some patients may experience non-typical tension-like headache.^38^

Other possible post-LP AEs include lower-back discomfort/pain, short-term numbness of the legs, nausea, vomiting, and dizziness; in rare cases, vasovagal symptoms such as hypotension or syncope have been reported.^26,29,31,32^ In the multicenter feasibility study conducted in a pooled cohort of 3868 patients, back pain was reported by 17% of patients; nausea, vomiting, or both by 2.5%; dizziness by 1.3%; and vasovagal episode by 0.5%.^29^ Another feasibility study involving 689 patients (mean [SD] age 62.4 [9.1] years) reported a similar incidence of back pain after LP (16.1%).^25^ Other studies performed in smaller populations reported a lower incidence of non-headache AEs compared to large-scale studies.^25,26,32^

A similar safety profile has been reported in a multicenter study investigating the feasibility of LP in the biological diagnosis of neurodegenerative diseases including Parkinson’s disease.^42^ The study enrolled 683 participants and reported an overall incidence of AEs after LP of 23%: more than two-thirds of events (68%) were rated as mild, while only 5.6% were rated as severe. The most common AEs were headache (13%) and lower-back pain (6.5%). Interestingly, both AEs proved to be significantly more frequent in healthy control individuals and patients with other neurological disorders than those with Parkinson’s disease.^42^ Similar findings were reported in the multicenter feasibility study, in which MCI or AD diagnosis was associated with lower risk of headache or back pain than for controls, after LP.^29^

Very rare (in <1 in 10,000 patients) but potentially disabling or fatal complications after LP include infection, cerebral or spinal hemorrhage, spinal epidural or subdural cerebral hematoma, and cerebral venous thrombosis.^31^ The risk of hemorrhage or thrombosis can be reduced by normalizing hemostatic factors such as platelet counts and coagulation parameters, including the international normalized ratio (INR) and/or the prothrombin time (PT). Anticoagulant medication (dicoumarol, warfarin, novel oral anticoagulants [NOACs] or low molecular weight heparin) should be reviewed before the procedure. Other diagnostic alternatives (i.e., PET) should be preferred over LP in a patient on antithrombotics. A temporary discontinuation may be considered case-by-case and performed only when there is good benefit/risk ratio, that is, lower risk of thrombosis during the discontinuation window. In addition, tight and regular monitoring of INR and/or PT changes, which generally fall or increase more slowly in older people than young adults, should be de deployed.^31,43^ The overall management of antithrombotics in the case of LP for suspected neurodegenerative disease follows the guiding principles of the elective (not in life-threatening conditions) procedure. For instance, the timing for withholding and resuming varies according to the pharmacological approach adopted to modulate the coagulation activity.^27,29,31–35,43^ In summary, an LP for the diagnostic work-up of a patient with AD who is on antithrombotics should be performed only when molecular imaging is not available and only after individualized and comprehensive estimation of all the potential risks of therapy discontinuation.

### 2.4 | The risk of AEs is not specialist-related

The risk of AEs does not significantly change when the procedure is performed by different health-care professionals. An analysis of 675 LPs carried out in prospective research studies reports that ≈95% of procedures (640/675) were performed by trained and qualified registered nurses (RNs) or nurse practitioners (NPs), and the rest (35/675) were performed by physicians.^34^ The study found that the incidence of post-LP headache is similar when the procedure was performed by RNs or NPs compared to when the procedure was performed by physicians (odds ratio [OR] 0.98 [95% confidence interval (CI), 0.23–4.26], *P* = .98).^34^ It is likely that the level of technical training on performing the LP procedure, rather than the specialty (physician vs. nurse), plays a major role in the risk of post-LP AEs. This key observation, consistent with experience from other centers that use NPs for LP, supports a more widespread implementation of LP in clinical outpatient practice.

### 2.5 | Patients’ perception of the procedure

The low rate of request for LP in the AD diagnostic work-up in some countries may be related to patient perception of the procedure. When patients are informed about the safety of the LP procedure, their perception is generally positive.^44^ Such patient education can be supported by available informative videos.^36^ One study enrolling 538 participants aged ≥65 years investigated older adults’ attitudes toward enrolment of non-competent patients with AD (i.e., patients who are demented and are not competent to provide informed consent) in research that does not present potential benefit to patients, such as exploratory studies on biomarkers; 75% of individuals interviewed declared that they would be willing to be enrolled in AD research using LP even though it does not offer treatment benefit to the patients.^30^

## 3 | PSYCHOGENIC FACTORS ON THE INCIDENCE OF POST-LP COMPLICATIONS

When discussing the frequencies of AEs such as headache and back pain after LP, a possible confounder is that almost all studies were unblinded. They were based on patients in a clinic undergoing LP as part of the diagnostic assessment, after which the patients were actively asked whether they had any AEs. This study design introduces a risk for bias, with overestimation of the frequencies of complications. For example, one study used a double-blind design to investigate complications after LP.^45^ The procedure was performed on 100 healthy volunteers; one group of 50 participants had a standard LP, while the other group had a sham LP with the needle inserted at the same place in the lumbar region, but not far enough to reach the subarachnoid space. Complications were reported by the volunteers, and evaluated by another physician, without knowledge of group assignment, and both led to believe that a standard LP had been performed. The incidence of headache after the procedure was not statistically different between groups, but post-LP headache was more common for those who expressed concern about this complication, suggesting that psychogenic factors play an important role in post-LP headaches.^45^ Similarly, in the multicenter LP feasibility study,^29^ anxiety was an independent risk factor for post-LP headache.

## 4 | RECOMMENDATIONS FOR REDUCING THE RISK OF POST-LP ADVERSE EVENTS

**FIGURE 2:**
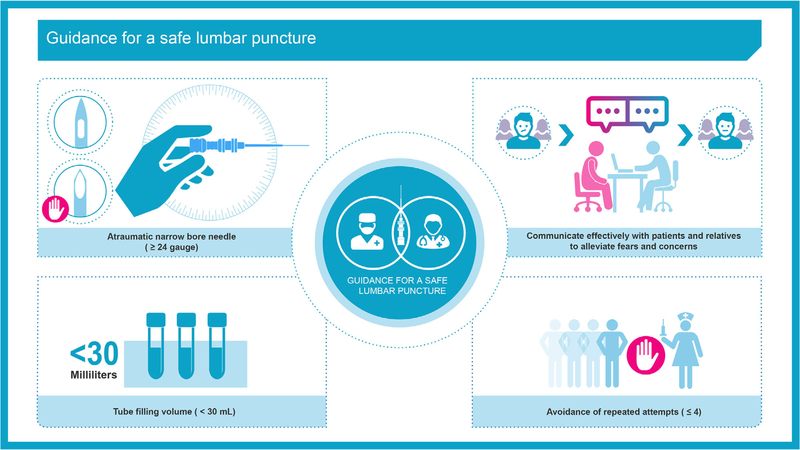
Guidance for a safe lumbar puncture. Expert consensus recommendations for reducing the risk of adverse events after lumbar puncture in patients with neurological diseases^29,31^

Expert consensus recommendations aimed at reducing the risk of AEs after LP in patients with neurological diseases have been published (Box 1) and are summarized in Figure 2. These recommendations are based on a systematic literature review on LP needle characteristics and post-LP complications, data from the multicenter LP feasibility study,^29^ and discussions with participants within the Joint Programme Neurodegenerative Disease Research Biomarkers for Alzheimer’s Disease and Parkinson’s Disease and Biomarkers for Multiple Sclerosis consortia.^31^

The consensus recommendations address two issues: (1) technical aspects of the procedure that contribute to a favorable safety profile, and (2) strategies for patient stratification based on risk factors for AEs. These recommendations, coupled with compliance with the criteria for indications of LP in individuals with suspected AD pathophysiology, offer three-part guidance for performing LP with maximal clinical confidence in the context of AD diagnostics. The most important risk factors for post-LP AEs are reported in Table 3. Below we reiterate and expand on some of the key points from the consensus recommendations.^31^

### 4.1 | Technical aspects of the LP procedure that contribute to a favorable safety profile

Characteristics of needles, such as the design, length, and diameter, are important technical aspects influencing the safety of LP. In general, the choice of the needle for a patient depends on the patient’s age and weight and the purpose of the procedure. The selection of a specific type of needle should aim to minimize discomfort and the risk of complications (Table 4).^31^

The use of a conventional cutting-bevel needle, rather than an atraumatic (blunt, “bullet” tip) needle, is an important procedure-related risk factor for post-LP headache. In a meta-analysis of 110 randomized controlled trials involving 31,412 patients, rates of post-LP headache associated with conventional and atraumatic needles were 11.0% and 4.2%, respectively (relative risk 0.40 [95% CI, 0.34–0.47], *P* < .0001).^46^ Based on the consistently favorable safety profile of atraumatic needles compared to cutting-bevel needles, their use has been recommended in multiple guidelines addressing neurological practice.^47–50^ The main drawback of atraumatic needles is the increased risk of procedure failure in specific categories of patients, such as those with severe obesity or primary/secondary spine deformity.^31,46,51^

Needle diameter (thickness) plays a major role in the risk of post-LP complications. Most studies recommend small-bore needles (≥24 gauge), which are associated with lower rates of headache, back pain, and discomfort, as well as a lower risk of blood contamination and CSF leakage.^31^ Small-bore needles are associated with slower CSF drip rate, making the sampling time longer.^31^ The time of CSF collection, however, is not a risk factor for post-LP AEs, and for diagnostic purposes, <1 millilitre (mL) is needed. The smallest needles (27–29 gauge) and the large-bore needles (≤22 gauge) are generally not recommended.^31,52^

Standard-length needles (70–90 mm) are used in adults, although longer needles may be needed in obese patients.^53,54^ The use of longer needles makes the procedure more difficult because such needles are more flexible and thus have a tendency to divert off track.^31,55^ This may necessitate multiple attempts at LP, causing local swelling, bruising, or muscle spasms, and increasing the risk of back pain.^31,55^ In the multicenter feasibility study, compared to individuals for whom the first attempt of LP was successful, the risk of back pain was approximately doubled when two to four attempts were required (OR 2.1 [95% CI, 1.7– 2.7]), and increased five-fold when five or more attempts were needed (OR 5.4 [95% CI, 2.9–10.2]).^29^ For this reason, it is recommended that no more than four attempts at dural puncture be made.^31,55^

Active withdrawal of CSF though a syringe reduces the sampling time but is associated with a greater risk of post-LP headache,^29^ and as such gravity flow removal is recommended compared to active CSF withdrawal.^56^ The volume of CSF withdrawn (<5mLvs.>12 mL, tested up to 30 mL) had no significant effect on the risk of post-LP headache or back pain.^29,32,34^ In an observational AD study, withdrawal of up to 30 mL of CSF had no adverse effect in terms of complication rates,^57^ and this volume is therefore recommended as an acceptable maximum.^31^

LP can be performed with the patient in either a lateral recumbent (supine) or sitting position.^34,57^ The preferred position depends on the physician and the patient’s condition.^31^ There is some evidence suggesting that the sitting position during LP might be associated with a higher risk for severe headache^29^ or immediate post-procedural headache.^57^

Evidence suggests that prolonged rest by lying down on a bed after LP is not associated with lower incidence of AEs compared to immediate mobilization.^58,59^ A systematic review of 24 randomized controlled trials shows that lying down shortly after the procedure has no significant effect on the risk of severe post-LP headache compared to immediate mobilization (relative risk 0.98 [95% CI, 0.68–1.41). A study conducted in 70 patients attending a neurology clinic reports no significant difference in rates of post-LP headache between participants who laid down for 1 or 4 hours after the procedure.^58^

### 4.2 | Patient stratification according to risk of adverse events

Health-care providers could stratify patients according to individual risk factors for post-LP AEs or severe complications (Table 3), thereby optimizing safety, time, resources, and costs of the procedure.

Younger age appears to be the most important patient-related factor affecting the risk of both post-LP headache and lower-back pain.^31^ In the multicenter feasibility study, the risk of typical post-LP headache in patients >65 years of age was 32% lower than in younger patients (OR 0.68 [95% CI, 0.46–1.00]), and the risk of lower-back pain was 44% lower (OR 0.56 [95% CI, 0.48–0.65]).^29^ Similar results were obtained in another feasibility study, involving 689 patients across three memory clinics.^25^ Increased age was associated with lower risks of any headache (OR 0.93 per year [95% CI, 0.91– 0.96]), typical post-LP headache (OR 0.94 per year [95% CI, 0.92–0.96]), and severe headache (OR 0.92 per year [95% CI, 0.87–0.97]).^25^ The negative association between risk of post-LP headache and age is supported by studies conducted in patients with different neurological disease and using different protocols.^57,60–62^ Furthermore, the dementia syndrome is associated with an overall reduced risk of post-LP headache. In the multicenter feasibility study, individuals with MCI and dementia had lower risks of complications than individuals with normal cognitive function (Table 2),^29^ and similar results have been reported in a longitudinal study involving 273 participants.^61^ A low incidence of post-LP headache (2%) has also been reported in an uncontrolled study of 395 patients with dementia.^63^ Such findings suggest that the routine use of LP in older patients with appropriate indications for the evaluation of MCI/dementia should not raise significant safety concerns.

In terms of sex, several studies reported that post-LP headache is more common in women than in men, and especially in women ≤40 years of age.^48,60,64–67^ However, the multicenter feasibility study, which was a large investigation performed in the aging population within memory clinics to define independent risk factors, found no evidence of a sex difference in older adults in the incidence of headache or back pain after LP.^29^

In a review of 675 LPs performed in prospective research studies, the risk of post-LP headache was significantly greater in participants with a body mass index (BMI) ≤25.0 kg/m^2^ than in those with a higher BMI (OR 3.3 [95% CI, 1.5–7.0], *P* = .001).^34^ Another study involving 239 patients undergoing diagnostic LP found that BMI had no effect on the incidence of post-LP headache; however, postural headaches tended to develop and resolve more slowly in women with the highest BMIs compared to those with lower BMIs.^68^

Fear of the procedure is an important modifiable risk factor. In the multicenter feasibility study, patients were questioned before LP to identify any such fears or a relevant medical history of headache.^29^ Compared to patients who reported no concerns, patients who described themselves as “very worried” were at significantly higher risk of non-specific headache (OR 2.01 [95% CI, 1.39–2.91]) or back pain (OR 1.41 [95% CI, 1.12–1.78]).^29^ Similarly, a history of headache was found to be an important risk factor for typical post-LP headache; the ORs for mild or severe headache in participants with a history of headache, compared to those with no such history, were 1.8 (95% CI, 1.4–2.6) and 2.7 (95% CI, 1.9–3.7), respectively.^29^ One study reported that post-LP headache was significantly more common in individuals with limited previous experience of the procedure (≤2 previous procedures) than in those with more experience (OR 2.1 [95% CI, 1.1–4.1], *P* = .03).^34^ In addition, a prospective study reported that LP is safe when performed in patients with Down syndrome to investigate AD.^69^

Identification of such patient-related risk factors can help to identify patients at increased risk of post-LP headache and other AEs and provides an opportunity to inform and reassure patients and caregivers before and during the procedure. Careful provision of information and reassurance of the patient are essential to reduce the risk of complications.^36^ Appropriate steps should be taken during the procedure to minimize anxiety and discomfort.

### 4.3 | Indications for use of CSF biomarkers in the diagnosis of AD and pre-procedure work-up: guidelines from international consortia

While the safety of the LP procedure is well established, it is critical that the procedure is applied according to guidelines for appropriate use and unnecessary or inappropriate procedures are avoided. The consensus guidelines for LP in patients with neurological diseases emphasized the importance of a detailed work-up to exclude potential contraindications for LP, such as space-occupying lesions with mass effects, coagulopathies, congenital spine abnormalities, and skin infections at the puncture site.^31^ Clinical neurological examinations should be performed to exclude space-occupying lesions, posterior fossa masses, or Arnold-Chiari malformation. Brain magnetic resonance imaging or computed tomography should be carried out in patients with abnormal clinical neurological findings, reduced consciousness, a relevant history of central nervous system disease, or recent seizures. The pre-procedure assessment should include confirmation of an adequate platelet count (>40 × 10^9^/L) and coagulation status (international normalized ratio <1.5), and exclusion of coagulopathies and uncorrected bleeding diathesis.^31^

For the use of LP in AD diagnostics specifically, an international workgroup convened by the US Alzheimer’s Association developed recommendations for the appropriate use of LP in the diagnostic work-up of AD, identifying six appropriate and eight inappropriate uses (Table 5).^33^ The aim of these guidelines is to standardize and optimize decision-making across general and specialist practitioners.

Of note, while these recommendations and guidelines are based on extensive existing data in large patient populations, most studies have been conducted in Western countries, and whether the findings can be generalized to other populations, such as those in Asia, requires further investigation. In addition, currently there are still significant regional differences in clinical practice, possibly due to perceptions. For example, physicians in Japan prefer post-LP bed rest for an hour, and they hesitate to draw >10 mL CSF. Regional observational studies should be performed when necessary to investigate perceptions as well as the risk factors for LP-associated AEs, which could generate medical evidence to inform the diagnostic work-up in specific populations.

## 5 | EMERGING TECHNIQUES AND INNOVATIONS IN LP

Because the number of attempts is a risk factor for AEs, there is a clear rationale to optimize the procedure. A growing trend during the past two decades has been the use of fluoroscopic guidance to aid LP in patients with particular clinical and anatomical conditions, such as obese patients or those with primary/secondary spinal deformities.^70–72^ Intermittent-pulse fluoroscopy can be used to identify the appropriate site for the LP and to monitor the position of the needle until it reaches the subarachnoid space. This approach can increase success rates and reduce rates of traumatic LP.^70–72^ However, the success rate is still largely operator-dependent.^70^ For example, in a retrospective review of 1489 bedside procedures and 723 fluoroscopy-guided procedures, rates of traumatic LP varied from 0% to 24% depending on the operator.^73^ Several recent studies suggest that “phantoms” or virtual simulations can be useful training aids to increase confidence in less experienced health-care providers.^74–76^ Such techniques are under development and validation, and hold the potential to inform and optimize procedural planning, anatomical guidance, and safety of LP in an individualized fashion.

The increase in the use of fluoroscopy-guided LP reflects the evolution of fluoroscopy into a sophisticated technology with advanced 3D imaging.^77^ 3D fluoroscopy can be useful in guiding LP in cases in which there are barriers to the bedside attempts.^78^ Additional technological advancements are expected to further facilitate the use of fluoroscopy to guide LP. In particular, the combination of CT with 3D fluoroscopy reduces the radiation dose required for fluoroscopy without compromising the spatial resolution.^79^

To circumvent the X-ray exposure, ultrasonography-based LP techniques are under development.^80,81^ This technique can provide additional information to facilitate needle placement that is not available from physical examination, particularly in obese patients or those with spine deformity.^81^ The clinical utility of ultrasound-based LP techniques has been demonstrated in a number of randomized controlled trials, and a meta-analysis of these studies reported higher procedural success rates with ultrasound-guided LPs compared to landmark/palpation-guided LPs (OR 2.1 [95% CI, 0.66–7.44]).^82^

Based on the accumulating evidence,^83,84^ recommendations for the use of ultrasonography in adults undergoing LP published by the US Society of Hospital Medicine propose the use of ultrasound for site selection to reduce the number of needle insertion attempts and needle redirections.^81,85^ Ultrasonography has the advantage of being applicable at the bedside, avoiding the need for a fluoroscopy suite.

A more advanced form of ultrasound-assisted LP is real-time ultrasound using a needle guidance system (NGS).^86^ In a randomized crossover study comparing real-time, NGS-assisted LP with standard pre-procedure ultrasound in a group of 24 medical students learning to perform the procedure on a gel phantom, NGS guidance was associated with a significantly greater number of successful punctures per participant compared to pre-procedure ultrasound (5 [interquartile range (IQR), 3.3–5.0] vs. 3 [IQR, 1.3–4.0], respectively, *P* = .005). Importantly, there was also a reduction in performance time (118 seconds vs. 80.6 seconds, respectively, *P* < .001), and 23 of 24 participants reported that they preferred the real-time, NGS-assisted LP.^86^

Traditionally, physicians have learned to perform LP through unstructured observation and supervision during medical training. Training can be improved by leveraging video-based or simulation-based training (using either phantoms^87^ or virtual-reality devices), which are increasingly being evaluated and adopted in adult neurology settings.^72–76,88–93^ Studies have consistently found that such training aids are effective and can improve operator confidence, an important finding considering that medical students or junior doctors often report a lack of confidence in performing the procedure,^94,95^ and operator stress has been associated with reduced patient confidence in the operator and an increased risk of post-LP headache in patients.^96^ Evidence that these benefits can translate into improved performance in clinical practice comes from a study involving 110 junior doctors with no previous experience performing LPs.^97^ Participants who received goal- and learner-centered video training showed better performance in a simulated ward setting than those who received traditional instruction.^97^ Effective training may confer an additional safety benefit to the patient because there is evidence that procedure-specific training may increase the use of atraumatic needles by junior doctors.^98,99^

## 6 | POTENTIAL CHALLENGES TO ROUTINE LP IN AD CLINICAL PRACTICE

Given the increasing demand for AD diagnosis, especially in the advent of a DMT, models and projections based on the current landscape indicate that existing health-care systems and related infrastructures available today do not have the capacity to accommodate such a demand for large-scale biological diagnostic assessment.^8,9^ This would result in long wait times and create unnecessary delays in therapeutic decision-making that are ultimately detrimental to patient health.

As described above, considering the safety, tolerability, and acceptance of LP for assessment of CSF AD biomarkers, it is very likely to become a key part of the AD patient journey. Besides the necessity of medical education of health-care providers about recommendations and guidelines for optimal practice of LP, resource and time constraints, low reimbursement rates, and provision of proper training are other challenges hampering the widespread use of LP in AD.^29^

Time and resource constraints represent major barriers to the use of LP for the diagnosis of early AD because alternative use of health-care provider time could be more convenient for a single hospital or neurology clinic. This issue is particularly relevant in high-volume centers that have competing needs for resources. For example, tertiary hospitals are likely to have limited outpatient procedure rooms, and LP is considered a time-consuming procedure. Tertiary hospitals may also have a limited number of standard outpatient rooms with enough space to perform LPs as these are designed for 10 to 20 patient consultations per session.

The time-effectiveness of LP may be improved by avoiding or eliminating procedures that are not proven to generate clinical benefit. For example, optimizing the time of post-LP rest in a hospital or outpatient setting is essential from a system readiness perspective. If the existing evidence that there is no significant clinical benefit with post-LP rest was corroborated, the overall time of LP could be drastically cut, thus facilitating patient turnover. Furthermore, with appropriate training, LP can be performed safely and effectively by nursing staff,^100^ thus reducing the demand for physician time and optimizing time and resource investment.^101^ Regardless of specialization, the personnel that perform LP should have sufficient technical training and practice, and ideally perform the procedure in a frequent and consistent manner, rather than an ad hoc and sporadic manner.

From a health-care—system resource perspective, the high medical costs for patients with AD, compared to cognitively normal individuals or those with MCI, are due primarily to high inpatient costs rather than the costs of diagnosis.^102^ Furthermore, several health economic studies suggest that CSF biomarker analysis is likely cost-effective. For example, one study found that the cost-effectiveness of CSF biomarker analysis depends on the pre-test prevalence of AD; in the scenario of patients referred to memory clinics with memory impairment who do not exhibit neuroimaging evidence of medial temporal lobe atrophy, pre-test prevalence of AD is relatively high (may exceed 15%), and CSF biomarker analysis was deemed cost-effective. As such, the study concluded that biomarker analysis should be considered for adoption in high-prevalence centers.^103^ Other studies found that detecting AD in patients with MCI using CSF biomarkers is cost-effective for disease prediction, progression monitoring, and diagnostic/therapeutic decision-making.^104–106^ Furthermore, a study estimated that in the UK, although the annual costs of additional amyloid PET scans or CSF tests are significant (100,000 extra amyloid PET scans and 100,000 extra CSF tests at £113 million and £48 million, respectively), they are rather modest compared to the likely market price of future DMTs or to the costs of inaccurate diagnosis.^107^ In addition, costs for performing LP may have been overestimated in the study, and were, for example, based on first-year costs for extensive training (estimated to be 80% of a 1-year salary) plus an additional full-time employment of nurses doing two LPs per day and having no other tasks, and may likely be substantially lower in a more streamlined scenario. Health systems in different countries/regions vary significantly, and so do cost-effectiveness measures, making it necessary to determine the cost-effectiveness in each local system. Nevertheless, it is expected that the cost-effectiveness associated with CSF biomarkers will significantly increase if an effective DMT becomes available.^106^

Another barrier is that in the absence of an effective treatment, patients and their families may hesitate to pursue a diagnosis,^108^ and health-care providers may have reservations about performing a procedure that is not without potential AEs and that may not change clinical management significantly. Thus, it is critical to educate patients and health-care providers about the importance of having an early diagnosis of AD.

Good communication between physicians and patients and their families is needed to overcome terminological misunderstandings and alleviate anxiety for the procedure. For example, the Japanese term for LP is “youtsui-senshi” and bone marrow aspiration is “kotsuzui-senshi.” Because of the similarity between the two terms, patients and their families might misunderstand LP as bone marrow aspiration, which is typically painful, leading to reluctance to undergo a LP. Anxiety has been identified as an independent risk factor for post LP headache;^29^ it is therefore conceivable that informing and reassuring patients about the procedure may help to increase the willingness and preparedness to undergo the procedure as well as decrease AEs. Informative videos could help improve the knowledge of patients and reduce their fears.^36^ A recent study involving 851 individuals who had previously indicated unwillingness to be contacted for research involving LP reported that those who received gain-framed video education (i.e., emphasizing the proportion of individuals free of AEs) had 67% higher odds of changing their response compared to those who received loss-framed video education (i.e., emphasizing the portion experiencing AEs), indicating that message framing is important for developing optimal patient education.^109^

Reimbursement is a critical “system readiness” barrier. While the expenses of performing LP procedures have been covered—to varying degrees—in the reimbursement frameworks in most countries, overall they tend to be low; the expense of CSF biomarker assays is largely not covered in reimbursement systems. For example, in the United States in 2020, the physician Medicare payment for a LP is officially listed as $143.28, down from $152.09 in 2019.^110^ In a retrospective analysis of 211 LPs performed at a single US center in 2017, the total billable cost of the procedures was $80,469, but the amount reimbursed was only $13,004.^111^ Moreover, of the 93 cases performed under fluoroscopic guidance for which a separate billing code was added, Medicare paid an average of $41. Thus, in some cases, reimbursement does not reflect the actual cost of the procedure.^111^ In Japan, reimbursement for the LP procedure and phospho-tau measurements combined is ≈$90 and physicians often do not believe that this fairly compensates for time and resources. In Europe, obtaining adequate reimbursement for the costs of CSF analysis can be challenging. A European survey of dementia specialists found that approximately half of the countries represented did not reimburse the cost of CSF analysis.^101^ One reason for this may be the lack of expert consensus guidelines on the use of CSF analysis for the diagnostic assessment of dementia, which leads to inconsistencies in practice and a lack of standardization of reimbursement.^101^ In certain Asian countries including Singapore, patients have limited medical insurance reimbursement for outpatient procedures.

**FIGURE 3:**
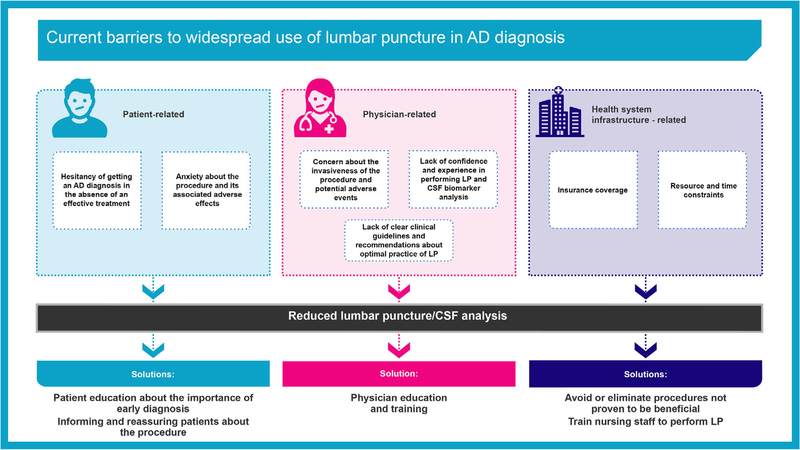
Current barriers to widespread use of lumbar puncture (LP) in Alzheimer’s disease (AD) diagnosis. Factors negatively influencing the widespread use of LP in AD diagnosis include patient-related, physician-related, and health system infrastructure-related barriers.^8,9,29,101,108^ CSF, cerebrospinal fluid

An overview of factors negatively affecting the widespread use of LP in AD is shown in Figure 3.

## 7 | CONCLUSIONS

After decades of research and considerable learning from hundreds of clinical trials, the field of AD is entering an era of accelerated development whereas a number of promising DMTs are on the near-term horizon. In addition, with the increase in longevity and population aging around the world, the existing health-care systems are facing an unprecedented challenge in timely diagnosis and management of patients with AD. It is imperative for the current health-care systems to improve and evolve to accommodate the increasing demand for AD diagnosis, and in the advent of DMTs being approved, the timely access, appropriate use, and affordability of costs incurred with DMTs.

Biomarkers are playing an increasingly essential role in guiding the diagnosis and therapeutic decision-making in AD. Compared to other well-validated biomarker modalities, CSF biomarkers represent a cost-, time-, and resource-effective approach with the potential to improve clinical practice in AD globally. However, their implementation in clinical routine is being delayed by several remediable factors. Available evidence from large patient populations shows that LP is a safe procedure that can be readily incorporated into clinical research and practice. By aligning the appropriate use of LP in the diagnostic work-up of AD to international LP consensus guidelines—with attention to both minimization of procedural risk factors and enabling patient stratification according to individual risk of AEs—very low rates of clinically significant AEs related to LP can be achieved. The requirement for LP should not, therefore, constitute a barrier to widespread use of CSF biomarkers in both clinical trials and medical practice for AD, as the need for a biopsy is not a barrier to diagnose and treat cancer. Emerging techniques and innovations in LP, such as the incorporation of fluoroscopic guidance and bedside ultrasonography when necessary, can further contribute to the safety and feasibility of the procedure in the uncommon scenario in which the regular procedure is not appropriate.

While CSF biomarker analyses have the potential to become a critical component of the globally accessible next-generation AD patient journey, significant variability has been documented in CSF biomarker measurements, which represents a major hurdle for its widespread use in clinical decision-making.^22,112,113^ From a methodological standpoint, pre-analytical and analytical protocols for CSF collection and handling, assay development, and biomarker analyses in clinical laboratories need to be harmonized.^22,112,113^

Pre-analytical workflows and analytical protocols should be harmonized and then standardized at a global scale to reduce inter-study/inter-site variability and eventually accelerate the biomarker development program, that is, discovery, analytical/clinical validation, and qualification. Pre-analytical factors encompass: different types of tubes, time and temperature before storage, storage temperature and length, repeated freeze/thaw cycles, among others, and consensus and harmonized preanalytical protocol for CSF handling have been developed to reduce variability and facilitate reproducibility in CSF biomarker measurements across studies and laboratories.^114,115^

In this regard, several international consortia and working groups such as The International Federation of Clinical Chemistry and Biomedicine Working Group on CSF-Proteins (WG-CSF) and The Alzheimer’s Association International Society to Advance Alzheimer’s Research and Treatment (ISTAART), as well as the European Union-North American Clinical Trials in Alzheimer’s Disease Task Force (EU/US CTAD Task Force) are intensely supporting the development of field-wide consensus on the understanding and control for preanalytical variables on the final biomarker concentrations.^22,113^ A unified pre-analytical protocol for the core AD CSF biomarkers (A*β*, total tau, and phosphorylated tau) has recently been devised,^116^ providing practical recommendations to overcome the issue of potential variability in clinical practice.

To follow, the validation steps will be facilitated by (1) widely accepted general requirements for the competence of testing and calibration laboratories and (2) developing standard operating procedures (SOPs) stemming from international consensus working groups.^117^ The ideal assay for large-scale diagnostic practice should fulfil an acceptable balance among the following factors: good accuracy, reproducibility, accessibility, and availability for larger-scale investigations; operability and low expert operator-dependency; and limited demand on cost and resource.

Different analytical platforms and assays vary in the absolute levels in the readout, resulting in different cut-offs (i.e., thresholds to identify pathology); it is important that investigators who use the same platform or assay for CSF analysis work together toward harmonized analytical protocols and globally accepted cut-offs. The recent development of fully automated assays provides the basis for the development of globally replicable and accepted cut-off points. Fully automated assays have already shown good analytical performance, including limit of quantitation, lot-to-lot comparability, repeatability, coefficients of variation, and estimated total reproducibility.^118–121^ These assays have now also been re-calibrated against the available certified reference materials (CRMs), to ensure the equivalence of results across methods and platforms, and to solve the issue of differences in absolute levels.^122^

Beyond global methodological standardization, flexible adjustment of biomarker cut-offs according to relevant biological factors—including age, sexual dysmorphism, and apolipoprotein E genotype—can improve test accuracy and data interpretation.^123,124^ In addition, the range around cutoff values within which there is little change in predictive performance for decline of memory, cognition, and function should be defined.^120^

The use of LP to enable CSF biomarker analysis in individuals at risk of or with early clinical features of AD offers the opportunity to perform a single test and attain the entire AT(N) system profile of the patient. A CSF-based AT(N) system can be instrumental for several contexts of use, including diagnosis, prognosis, screening for enrolment in clinical trials, and assessment of target engagement and treatment efficacy. Optimized use of CSF biomarker analysis could enable widespread early detection and management of patients suffering from AD globally. In this context, a cross-functional collaborative effort across academia, the pharmaceutical and biotech industry, the health-care community, patient advocacy groups, and regulatory agencies is essential to construct the appropriate practice paradigm of LP and CSF biomarker analysis and establish it as an important component within the new-generation patient journey, ensuring early disease detection and timely therapeutic decision-making in AD.

## Figures and Tables

**TABLE 1:** The amyloid/tau/neurodegeneration (AT[N]) research framework for AD^7^

	Cognitively unimpaired	Mild cognitive impairment	Dementia
A^−^T^−^N^−^	Normal AD biomarkers, cognitively unimpaired	Normal biomarkers with MCI	Normal AD biomarkers with dementia
A^+^T^−^N^−^	Preclinical AD pathological change	AD pathological change with MCI	AD pathological change with dementia
A^+^T^+^N^−^ A^+^T^+^N^+^	Preclinical AD	AD with MCI (prodromal AD)	AD with dementia
A^+^T^−^N^+^	AD and concomitant suspected non-AD pathological change, cognitively unimpaired	AD and concomitant suspected non-AD pathological change with MCI	AD and concomitant suspected non-AD pathological change with dementia
A^−^ T^+^N^+^ A^−^ T^−^N^+^ A^−^ T^+^N^+^	Non-AD pathological change, cognitively unimpaired	Non-AD pathological change with MCI	Non-AD pathological change with dementia

Abbreviations: A/T/N, amyloid beta deposition, pathologic tau, and neurodegeneration; AD, Alzheimer’s disease; MCI, mild cognitive impairment.

+ , positive for biomarker based on dichotomous cutoff value.

− , negative for biomarker based on dichotomous cutoff value.

**TABLE 2:** Key studies of LP safety in patients treated in memory clinics, or healthy volunteers

Study	Participants	Procedural details	AE profile	Comments
BIOMARKAPD multicenter LP feasibility study^29^	3868 patients attending 23 memory clinics: - Female: n = 3855 (49.9%) - Mean (SD) age: 66 (11) years - Mean (SD) MMSE: 25 (5) Diagnosis: - Healthy individuals (SCI): n = 754 (19.5%) - MCI: n = 946 (24.5%) - AD: 1052 (n = 27.2%) - Other dementia: n = 478 (12.4%) - Psychiatric disorders: n = 167 (4.3%) - Neurological disorders: n = 215 (5.6%) - Other/unclear: n = 256 (6.6%)	Needle type: - Cutting edge: n = 2956 (83.1%) - Atraumatic: n = 560 (15.7%) Needle diameter: - ≤25 G: n = 982 (27.6%) - 23–24 G:n = 212 (6.0%) - 22 G:n = 1,129 (31.7%) - 21G: n = 309 (8.7%) - 19–20 G:n = 899 (25.3%) Patient position: - Lying: n = 1779 (50.0%) - Sitting: n = 1754 (49.4%) CSF collection: - Free flow/dripping: n = 2749 (77.3%) -Activewithdrawal: n = 661 (18.6%) Volume of CSF collected: - <5mL: n = 450 (12.6%) - 5–12 mL:n = 1761 (49.5%) - >12 mL: n = 1214 (34.1%)	Any AE: n = 1065 (30.8%) Headache: n = 649 (18.8%) - TypicalPLPH: n = 296 (8.6%) - Non-specific headache: n = 353 (10.2%) Back pain: n = 589 (17.0%) - Mild: n = 462 (13.3%) - Moderate or persistent (lasting several days): n = 127 (3.7%) Nausea and/or vomiting: n = 86 (2.5%) Dizziness: n = 45 (1.3%) Vasovagal collapse: n = 16 (0.5%) Severe complications: - Blood patch needed: n = 11 (0.3%) - Hospitalization needed: n = 23 (0.7%) - Emergency department visit, without hospitalization: n = 3 (0.1%) - Death: n = l^a^	• 513/649 headaches (79.0%) were mild (patient functioning normally) or moderate (impaired functioning, but hospitalization not required) in severity • 498/649 headaches (76.7%) resolved within ≤4 days; 165 (25.4%) resolved within <1 day • 248/649 headaches (38.2%) required no treatment; 379 (58.4%) resolved with analgesic medication • Factors associated with reduced risk of typical post-LP headache were: - Age >65 years (OR 0.68; 95% CI, 0.46–1.00) - Dementia (OR 0.84; 95% CI, 0.55–0.80) - Use of atraumatic needle (OR 0.39; 95% CI, 0.20–0.75) • Factors associated with reduced risk of back pain were: - Age >65 years (OR 0.56; 95% CI, 0.48–0.65) - MCI (OR0.72; 95% CI, 0.54–0.97) - Dementia (OR 0.74; 95% CI, 0.56–0.99)
Spanish multicenter study^25^	689 patients attending three memory clinics in Spain: - Female: n = 361 (52.4%) - Mean (SD) age: 62.4 (9.1) years Diagnosis: - Healthy controls: n = 239 (35.3%) - SCI: n = 142 (20.9%) - MCI: n = 127 (18.7%) - AD: n = 105 (15.5%) - Other: n = 65 (9.6%)	Needle type: - Cutting edge: n = 449 (65.2%) - Atraumatic: n = 240 (34.8%) Needle diameter: - 20G: n = 169 (24.5%) - 22 G: n = 520 (75.5%) Patient position: - Lateral decubitus: n = 481 (69.8%) - Sitting: n = 208 (30.2%) CSF collected by gravity dripping at all centers Mean (SD) volume of CSF: 9.8 (1.9)mL	Any AE: n = 248 (36.0%) Headache: n = 171 (24.8%) - Typical PLPH: n = 140 (20.3%) - Non-specific headache: n = 31 (4.5%) Back pain: n = 111 (16.1%) Dizziness or nausea: n = 27 (3.9%)	• Risk of headache reduced with age (OR 0.95; 95% CI, 0.93–0.97) per year • Fear of procedure associated with increased risks of PLPH (OR 2.02; 95% CI, 1.31–3.12) and back pain (OR 1.809; 95% CI, 1.17–2.78) • Incidence of back pain higher in women than in men (OR 1.95; 95% CI, 1.25–3.04) • Use of atraumatic needles associated with: - Lower incidence of AEs (OR 0.35; 95% CI, 0.17–0.37) - Lower incidence of PLPH (OR 0.28; 95% CI, 0.17–0.47) - Lower incidence of back pain (OR 0.38; 95% CI, 0.23–0.64) • Sitting position associated with increased risk of severe headache (OR 4.70; 95% CI, 1.68–13.1)
Peskind et al. 2005^32^	Participants enrolled in studies of CSF biomarkers: - Cognitively normal adults: n = 275 - Patients with MCI/AD: n = 67	All punctures performed with 24 G atraumatic needle Patient position: - Lateral decubitus: n = 281 - Sitting: n = 147 CSF withdrawn by syringe 87 participants underwent two LPs	Any AE: n = 47 (11.0%) Clinically significant AEs:^b^ n = 17 (4.0%) Mild headache: n = 19 (4.4%) Moderate headache: n = 6 (1.4%) Severe PLPH: n =4 (0.9%) Mild lower-back soreness: n = 11 (2.6%) Moderate lower-back soreness: n = 2 (0.5%) Vasovagal symptoms:^c^ n = 4 (0.9%) Mild nausea: n = 3 (0.7%) Others:^d^ n = 1 (4.3%)	• Risk of PLPH unrelated to age, sex, position during LP, volume of CSF collected, or duration of rest after procedure • Incidence of PLPH was significantly lower in participants with MCI/AD than in cognitively normal individuals (*P* = .03)
de Almeida et al. 2011^34^	477 research participants (675 LPs) at a single center Mean (SD) age: 42 (9) years Prior LPs: 0: n = 144 (30.2%) 1: n = 73 (15.3%) 2: n = 54 (11.3%) >2: n = 206 (43.2%)	All LPs performed using a 22 G atraumatic needle Median (IQR) volume of CSF collected: 13 mL (12–14)	PLPH: n = 38 (5.6%) Transient back pain and bleeding (number of cases not reported)	• 32/38 PLPHs resolved after rest, hydration, and over-the-counter analgesics; three required prescription analgesics, and one required a blood patch • Significant risk factors for PLPH were body mass index ≤25 kg/m^2^ (OR 3.3; 95% CI, 1.5–7.0) and ≤2 previous LPs (OR 2.1; 95% CI, 1.1–4.1)
Vilming et al. 2001^68^	239 patients undergoing diagnostic LP Female: n = 155 (64.9%)	All punctures performed using 20 G or 22 G needles	PLPH developed in 88/239 patients (36.8%)	• PLPH was significantly more common in females than in males (46% vs. 21%, respectively; *P* = .0003) • Severe PLPH was also more common in females (64% vs. 23%, respectively; *P* = .02) • Prevalence of PLPH was not related to age, weight, height, or body mass index • PLPH was significantly more common with 20 G needles than with 22 G needles (50% vs. 26%, respectively; *P* = .0002)
Vidoni et al. 2014^125^	525 patients enrolled in ADNI Diagnosis: - Non-dementia: n = 114 - MCI: n = 311 - AD: n = 100	Lumbar puncture performed with either cutting edge (n = 221) or atraumatic (n = 304) needles Needle diameter 18/20 G (n = 61),22G (n = 220), or 24/25 G (n = 244) CSF collected by either gravity drip (n = 214) or negative pressure (n = 300)	Incidence of PLPH: - Non-dementia: n = 3 (2.6%) - MCI: n = 21 (6.4%) - AD: n = 4 (4.0%)	• Incidence of PLPH was lowest with 24 G atraumatic needles (n = 3; 1.3%) • No significant difference in PLPH rates between punctures using gravity drip or negative pressure to collect CSF (6.7% vs. 3.7%, respectively)

Abbreviations: AD, Alzheimer’s disease; ADNI, Alzheimer’s Disease Neuroimaging Initiative; AE, adverse event; BIOMARKAPD, Biomarkers for Alzheimer’s Disease and Parkinson’s Disease; Cl, confidence interval; CSF, cerebrospinal fluid; IQR, interquartile range; LP, lumbar puncture; MCI, mild cognitive impairment; MMSE, Mini-Mental State Evaluation; OR, odds ratio; PLPH, post-lumbar puncture headache; SCI, subjective cognitive

a Caused by intracerebral hemorrhage due to oral anticoagulant treatment 2 days after LP.

b Moderate or severe headache, moderate lower-back soreness, vasovagal symptoms, and “other.”

c Pallor, diaphoresis, and transient hypotension with or without nausea.

d Moderate leg cramps accompanied by nausea.

**TABLE 3:** Risk factors for headache after LP^31^

Patient related	Procedure related
• Younger age • Female sex • Pasthistoryofheadache • BMI ≤25 kg/m^2^ • Less previous experience of LP • Fear of the procedure	• Use of a cutting-bevel needle rather than an atraumatic needle • Use of a large-bore (≤22 gauge) needle • Number of LP attempts • Active rather than passive withdrawal of CSF • Withdrawal of >30 mL of CSF • Sitting posture during procedure

Abbreviations: BMI, body mass index; CSF, cerebrospinal fluid; LP, lumbar puncture.

**TABLE 4:** Characteristics of needles used for LP^31^

Needle	Comparison	Advantages	Disadvantages
Design	Cutting bevel	• Penetration is felt through skin	• Increased complication rates • Requires more use of medications and medical assistance, resulting in higher costs
	Atraumatic	• Reduced complication rates • Decreased medical health-care costs due to fewer complications and less need for medical assistance and medications • Decreased traumatic taps	• Decreased flow rates, resulting in longer samplingtimes • More attempts and failures • Penetration through skin is difficult to feel
Length	Regular (70–90 mm)	• Use in adults	-
	Long (>90 mm)	• Use in obese patients	• Challenging approach
Diameter	Small (≥24 gauge)	• Reduced complication rates • Decreased pain and discomfort • Less risk of blood contamination • Requires less medical assistance and medications	• Decreased flow rates, resulting in longer samplingtimes • More failures • Requires training and practice
	Large (≤22 gauge)	• Increased flow rates • Shorter sampling times • Fewer failures	• Increased complication rates • Larger perforations • Greater risk of contamination

Abbreviation: LP, lumbar puncture.

**TABLE 5:** Clinical indications for appropriate use of LP and CSF analysis in the diagnosis of AD^33^

Appropriate indications for LP	Situations in which LP is *not* indicated
• Patients with SCD (cognitively unimpaired based on objective testing) who are considered to be at increased risk for AD • MCI that is persistent, progressing, and unexplained • Patients with symptoms that suggest possible AD • MCI or dementia with an onset at an early age (<65 years) • Meeting core clinical criteria for probable AD with typical age of onset • Patients whose dominant symptom is a change in behavior (e.g., Capgras syndrome, paranoid delusions, unexplained delirium, combative symptoms, and depression) and for whom AD diagnosis is being considered	• Cognitively unimpaired and within normal range functioning for age as established by objective testing; no conditions suggesting high risk and no SCD or expressed concern about developing AD • Cognitively unimpaired patient based on objective testing but considered by patient, family informant, and/or clinician to be at risk for AD based on family history • Patients with SCD (cognitively unimpaired based on objective testing) who are not considered to be at increased risk for AD • Symptoms of REM sleep behavior disorder • Use to determine disease severity in patients who already received a diagnosis of AD • Individuals who are *APOE ε4* carriers with no cognitive impairment • Use of LP in lieu of genotyping for suspected autosomal-dominant AD-mutation carriers • Autosomal-dominant AD-mutation carriers with or without symptoms

Abbreviations: AD, Alzheimer’s disease; APOE *ε*4, gene apolipoprotein E allele *ε*4; LP, lumbar puncture; MCI, mild cognitive impairment; SCD, subjective cognitive decline.

## BOX 1

Consensus recommendations to reduce the risk of adverse events after lumbar puncture for collection of cerebrospinal fluid (CSF).^29, 31^

- Evaluate potential contraindications.
- Identify patient-related risk factors.
- Place patient in the lateral recumbent position.
- Use an atraumatic narrow-bore (≥22-gauge) needle.
- Avoid multiple attempts (≤4).
- Passive rather than active withdrawal of CSF when feasible.
- Collect <30 mL of CSF.
- Communicate effectively to allay patient’s fears and concerns.

## RESEARCH IN CONTEXTS

1. **Systematic review**: In this review, we critically analyze the most recent and relevant evidence about the safety profile of lumbar puncture (LP) when applied to individuals with neurodegenerative diseases, particularly in groups most likely to be investigated and treated for Alzheimer’s disease (AD), based on data from >6000 participants in observational studies and/or attending memory clinics. In addition, we review the evidence-based clinical guidance and recommendations for optimized use of LP in individuals with suspected AD pathophysiology. Furthermore, we highlight new and emerging techniques of LP that hold the potential to further increase the operability, time-effectiveness, and eventually safety of the procedure. The authors reviewed the literature using PubMed, Google Scholar, and other sources.
2. **Interpretation**: Available evidence from large patient populations shows that LP is a generally safe procedure that can be readily incorporated into clinical research and practice. By aligning the appropriate use of LP in the diagnostic work-up of AD to international LP consensus guidelines—with attention to both minimization of procedural risk factors and enabling patient stratification according to individual risk of adverse events (AEs)—very low rates of clinically significant AEs related to LP can be achieved. The requirement for LP should not, therefore, constitute a barrier to widespread use of cerebrospinal fluid (CSF) biomarkers in both clinical trials and medical practice for AD.
3. **Future directions**: The review also contains an in-depth discussion about the challenges and barriers in global and regional health-care systems that need to be overcome along the path toward incorporating LP and CSF biomarker analysis in the diagnostic journey for patients with AD. Identifying and implementing solutions to address such barriers would be an important next step toward optimizing the next-generation AD patient journey.
